# Quantitative clinical assessment of wrist proprioception with stroke survivors

**DOI:** 10.1177/02692155251410469

**Published:** 2026-01-05

**Authors:** Yvonne YK Mak-Yuen, Thomas A Matyas, Kylee Lockwood, Leeanne M Carey

**Affiliations:** 1 Occupational Therapy, School of Allied Health, Human Services and Sport, 2080 La Trobe University, Bundoora, Victoria, Australia; 2 Neurorehabilitation and Recovery, 393884 The Florey, University of Melbourne, Heidelberg, Victoria, Australia; 3 Department of Occupational Therapy, 60078 St Vincent's Hospital Melbourne, Fitzroy, Victoria, Australia

**Keywords:** cerebrovascular accident, somatosensory, proprioception, standardised assessment, upper extremity, hand

## Abstract

**Objective:**

The aims of this study were to characterise proprioceptive impairment in individuals after stroke using the Wrist Position Sense Test (WPST) in a relatively large pooled sample, to re-establish the criterion of abnormality of the WPST, and to determine the sensitivity and specificity of a briefer test version for use in clinical settings.

**Design:**

Cross-sectional observation study with pooling of data across studies.

**Setting:**

Rehabilitation or outpatient settings.

**Subjects:**

Stroke survivors (n = 205) and neurologically healthy controls (n = 93) were assessed at baseline.

**Main measure:**

Wrist proprioception assessed using the WPST.

**Methods:**

Baseline data from stroke survivors and healthy controls assessed on the WPST was extracted from six studies. Raw data were pooled and analysed to determine an updated criterion of impairment and ability of a brief 10-trial version to detect proprioceptive impairment.

**Results:**

Proprioceptive impairment was common for the contralesional wrist (66%) and present in the ipsilesional wrist (21%). The new criterion of abnormality was established as 11.1^0^ average error. High sensitivity and specificity were found for the brief 10-trial version, with 85.3% sensitivity and 95.7% specificity.

**Conclusion:**

Clinicians can quantitatively and confidently identify proprioceptive impairment in the upper limb after stroke using either the original or brief version of the WPST. Routine use of this quantitative, standardised clinical assessment can contribute to improved identification, monitoring, and access to targeted intervention for proprioceptive impairment following stroke.

## Introduction

Stroke affects 17 million people annually worldwide and is a leading cause of disability.^1,2^ Upper limb sensory loss, including proprioceptive impairment, occurs in approximately 50% of stroke survivors,^3–6^ impacting motor recovery,^7^ functional outcomes,^4,8^ and quality of life.^9^ Proprioception is crucial for coordinating upper limb movements^10^ and impairment can hinder sensorimotor rehabilitation.^2,8,10^ While proprioceptive impairment can affect the entire upper limb, wrist proprioception is particularly important for functional recovery, as it contributes to safe and accurate positioning of the hand for reach, grasp, and manipulation of objects, especially when visual input is limited.^2^ Deficits in wrist proprioception impair everyday tasks like reaching for and using objects, which can limit independence and slow functional rehabilitation.^2,8,10^

Quantitative, standardised measures with good discriminative validity and sensitivity are critical to diagnose impairment, plan effective rehabilitation programs, monitor progress, and evaluate treatment effectiveness.^4,8,11^ However, clinical settings often rely on non-standardised measures with poor sensitivity.^12,13^ For example, using non-standard clinical testing, impaired proprioception at the wrist was missed in 77% percent of cases across affected and ‘unaffected’ hands, including moderate and severe impairment, compared to quantitative, standardised clinical testing.^14^ To effectively support recovery and facilitate return to meaningful activities, assessment tools must deliver targeted, quantitative results, accurately identify impairments, and be practical for use in clinical settings.

A range of assessments have been developed for clinical and research settings to identify proprioceptive impairment post-stroke^10,^^15–18^; however, their effectiveness varies, and some assessments lack validity, failing to measure the specific proprioceptive functions they intend to measure. One quantitative tool for assessing wrist proprioception post-stroke is the Wrist Position Sense Test (WPST).^3,14^ The WPST has the advantage that it is quantitative; uses standard test protocol; has good scale resolution; high retest reliability (r = 0.88 and 0.92); and norms to assist interpretation and good discriminative validity when used with adult stroke survivors.^3,19^ This test demonstrated ability to discriminate between impaired and unimpaired proprioceptive discrimination performance; eliminates the potential confounds of response matching via the other limb; and has alternative response modes for visual, movement or communication limitations post-stroke.^3^

Despite being designed for use with stroke survivors in clinical settings, the WPST remains underutilised in routine clinical practice – potentially due to perceived testing time demands and limited clinician confidence in interpreting results. Furthermore, existing normative data and cut-off scores for abnormality were derived from a relatively small sample of 50 stroke and 50 healthy controls,^3^ limiting the ability to confidently interpret scores and account for age and gender. With access to larger pooled datasets, there is now an opportunity to update these benchmarks. Updating normative standards and re-establishing criteria for abnormal performance will enhance the WPST's clinical interpretability and accuracy in identifying proprioceptive impairment post-stroke.

In the present study, we sought to pool baseline WPST data from six completed studies^3,^^19–23^ to:

- update normative standards and criteria of abnormality of the WPST;
- compare impaired WPST scores between the original 20-trial version and a briefer 10-trial version;
- determine the sensitivity and specificity of the 10-trial version for detecting somatosensory loss relative to the 20-trial version.

## Methods

### Study design and samples

Baseline WPST data from stroke survivors (n = 205) were pooled from six independent studies conducted by Carey and colleagues.^3,19–25^ The pooled database project was approved by Austin Health HREC/17/Austin/281 and La Trobe University Human Ethics Committees, Melbourne, Victoria, Australia. Raw data from paper-based or electronic files was obtained from the following studies: Discriminative Validity study^3^; Study of the Effectiveness of Neurorehabilitation on Sensation study^19^; Imaging Neuroplasticity of Touch study^20,21^; Connecting New Networks for Everyday Contact through Touch study^22,23^; additional testing linked with the National Institute of Health Toolbox study^24^; and the SENSe CONNECT Study.^25^ The data were pooled across studies because they shared similar participant characteristics and employed the same WPST and standardised protocol to assess proprioceptive discrimination ability.

Participants included those with a first stroke, with and without somatosensory loss, according to standardised clinical assessments. Participants were recruited from hospitals, rehabilitation centres, and the community, spanning from sub-acute to chronic phases of recovery. Inclusion criteria were similar across all studies, though there was some variation in criteria for time since stroke. Common inclusion criteria included the ability to understand two step instructions, medically stable, and able to provide informed consent. The Imaging Neuroplasticity of Touch and Connecting New Networks for Everyday Contact through Touch studies also required right-hand dominance, first stroke, and ability to undergo magnetic resonance imaging.^20,21^ The Study of the Effectiveness of Neurorehabilitation on Sensation study^19^ required participants to be at least 6 weeks post-stroke, while the Connecting New Networks for Everyday Contact through Touch study^22,23^ at least 12 weeks post-stroke. For the SENSe CONNECT trial,^25^ participants with more than one stroke were excluded to maintain consistency across studies. Exclusion criteria included having central nervous system dysfunction other than stroke, diagnosis of peripheral neuropathy, or presence of unilateral spatial neglect.

Data from healthy adult controls (n = 93) were pooled from the Normative/ Discriminative Validity study,^3^ the Connecting New Networks for Everyday Contact through Touch study,^22,23^ and additional testing linked with the National Institute of Health Toolbox study.^24^ These participants had no history of neurological dysfunction or upper limb somatosensory impairment and were not duplicated across studies.

Research therapists were trained in administration of the WPST and for intervention studies remained blinded to intervention conditions. Demographic information including age, sex, and hand dominance were collected from each participant's research file.

### Measure: Wrist Position Sense Test

**Figure 1.**
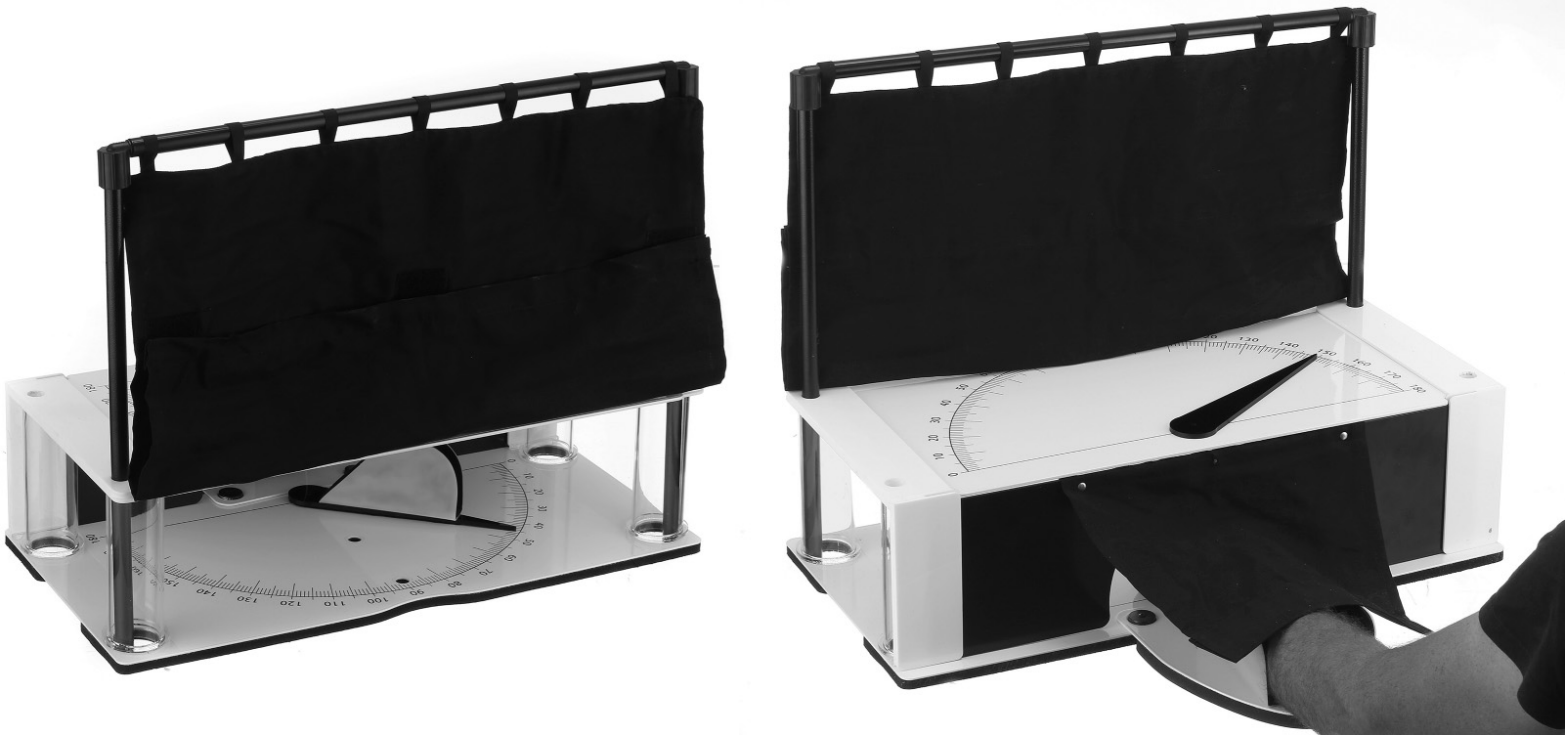
Wrist Position Sense Test.^26^

The WPST, developed by Carey et al., in 1996, is a standardised tool used to quantify one's ability to indicate their wrist position sense following an imposed wrist movement^3^ (see Figure 1). The participant, with vision occluded, matches 20 predetermined wrist positions in the flexion-extension range. The participant's hand is placed in a splint within a boxlike apparatus, and the assessor moves the wrist to defined test positions. Using a pointer on a protractor scale, the participant indicates their perceived wrist angle. Response scores are calculated as the average absolute degree of error (i.e. difference between actual and perceived wrist position) across 20-trials. Thus, a magnitude estimation, or direct scaling, approach is adopted to efficiently determine intact or impaired wrist position sense.

### Data analysis

Data analysis was conducted in two parts. In Part 1, we established updated normative standards and criteria for abnormality. Descriptive statistics were used to characterise WPST scores and included the key percentiles (5^th^, 25^th^, 50^th^, 75^th^ and 95^th^), frequency distributions, and range/spread of scores. Histograms compared scores between contralesional and ipsilesional hands of stroke participants and dominant and non-dominant hands of healthy controls. Regression models and bootstrap analysis examined the effects of age, hand dominance, and sex. The presence of impairment was identified relative to the criteria of abnormality defined using the 95^th^ percentile of neurologically healthy samples and confirmed against stroke distributions. Normative standards and criteria for abnormality were defined separately for the original 20-trial and abbreviated 10-trial versions to enable direct comparison across test formats. The confidence interval around the 95^th^ percentile and a zone of uncertainty around the criterion of abnormality was calculated using the standard error of measurement to guide interpretation of impairment. Quantile regression assessed the impact of age on the 95th percentile.

In Part 2, we evaluated the brief 10-trial version against the original 20-trial version. Scatterplots and linear regression were used to evaluate the association between the briefer 10-trial and original 20-trial test versions for the contralesional hands of stroke survivors.^27^ Contingency table analyses determined the sensitivity and specificity of the 10-trial version compared to the original 20-trial version,^27^ with sensitivity representing the ability to correctly identify impairment and specificity reflecting accuracy in identifying unimpaired performance.^28^ The first 10 trials were selected for the brief test version analysis, preserving a balanced distribution of wrist flexion and extension angles consistent with the original test. All quantitative analyses were conducted using Microsoft Office Excel 2016 and IBM SPSS Statistics (Version 28.0; IBM Corp., Armonk, NY).

## Results

### Participants

Data files of 205 stroke survivors (mean 56.1 years, standard deviation 14.5 years), pooled from six studies, were analysed. Most participants (84.9%) were right-hand dominant. Demographic characteristics of the pooled stroke sample are reported in Supplementary Table 1. Demographic data of 93 healthy controls (mean 51.5 years, SD 16.7 years), pooled from three studies, are also reported in Supplementary Table 1. Eighty-eight control participants (95%) were right-hand dominant.

### Updated normative standards and criteria of abnormality

Frequency distributions of average error scores of the 20-trial version for healthy participants (n = 93) were slightly skewed to the right for both dominant (0.70) and non-dominant hands (0.65). The scores ranged from 3^0^ to 14.5^0^ average error for dominant and 2.2^0^ to 12.8^0^ for non-dominant hands.

Investigation of age, sex, and hand dominance relationships with WPST 20-trial version scores for healthy adults revealed minimal changes in slope with increasing age, applicable to both dominant and non-dominant hand samples. Age accounted for less than 1% of the variance in test scores for both sides. The 95^th^ percentile confidence interval for the non-dominant hand [95% CI: −0.05, 0.10] fell entirely within the 95^th^ percentile confidence interval for the dominant hand [95% CI: −0.17, 0.12], with both intervals including the value zero, therefore failing to support a case for an age-adjusted or hand dominance-adjusted impairment threshold.

The combined 95^th^ percentile for males and females yielded an estimate of 11.16^0^ average error. Confidence intervals remained largely overlapped for males [95% CI: 10.22, 12.22] and were contained within the confidence interval for females [95% CI: 10.00, 13.13]. Thus, there is no evidence to support the use of separate impairment thresholds according to sex.

Normative standards and criterion of abnormality were defined for the original 20-trial and the briefer 10-trial versions (Table 1). Based on the combined dominant and non-dominant hand scores of the neurologically healthy samples, the 20-trial version exhibits a 95^th^ percentile criterion of abnormality at 11.1^0^ average error and the 10-trial version at 10.9^0^.

**Table 1.** Descriptive statistics and criterion of abnormality from combined dominant and non-dominant hands of the pooled healthy sample for WPST 20-trial and 10-trial versions.

WPST trial version	Mean Absolute error score	SD	Criterion of abnormality	CI for 95^th^ percentile		SEM	Zone of uncertainty for 95^th^ percentile	
			95^th^	LCI	UCI		LCI	UCI
20-trial	6.75	2.34	11.1	10.3	12.3	0.66	10.5	11.8
10-trial	6.27	2.41	10.9	9.8	12.5	0.93	10.0	11.8

*Note*: Criterion of abnormality is reported as the 95^th^ percentile. The zone of uncertainty for the criterion of abnormality is derived from the CI for the 95^th^ percentile and SEM.

WPST: Wrist Position Sense Test; SD: standard deviation; CI: confidence interval; LCI: lower CI; UCI: upper CI; SEM: standard error of measurement.

**Figure 2.**
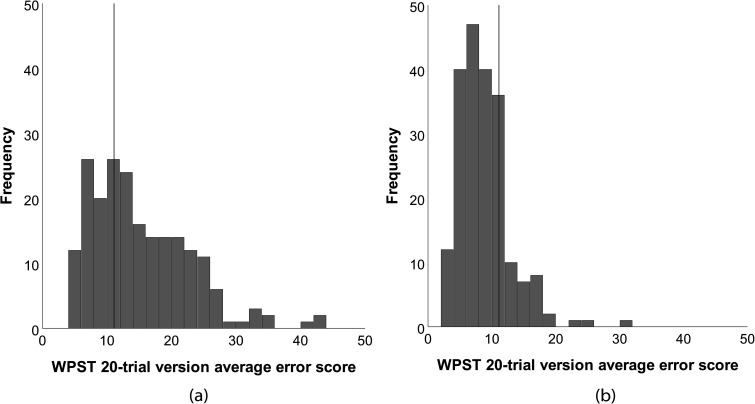
Frequency distributions of Wrist Position Sense Test (WPST) average error scores for (a) contralesional and (b) ipsilesional hands of stroke participants. The solid vertical lines represent the best estimate for the 95th percentile criterion of abnormality for the 20-trial version. Scores greater than 11.1^0^ average error indicate impairment.

The frequency distributions of average error scores for stroke participants (n = 205) were right skewed for both contralesional (0.99) and ipsilesional hands (1.62) based on the 20-trial version (Figure 2). Using the criterion of abnormality of 11.1^0^ average error, 66% of the total sample (136/205) were defined as having impairment in the contralesional hand. Scores ranged from 42.4^0^ average error, indicative of most severe impairment, to 4.3^0^ average error, well within the ‘healthy’ performance range.^29^ Impaired performance of the ipsilesional hand was also present in 21% (44/205) of the sample. Scores ranged from 31.0^0^ average error to 3.0^0^ average error.

Descriptive statistics, including score range and key percentiles, of 20-trial and 10-trial version scores for contralesional and ipsilesional hands are shown in Table 2. A median absolute error of 13.6^0^ on the contralesional side for the 20-trial version is considerably higher than the median absolute error found for the ipsilesional side (8.1^0^) and for healthy controls (6.0^0^). Median mean absolute error ranged from 11.7^0^ to 13.6^0^ across trial versions for the contralesional hand and from 7.5^0^ to 8.1^0^ for the ipsilesional hand.

**Table 2.** Descriptive statistics for the contralesional and ipsilesional hand of stroke survivors based on WPST 20-trial and 10-trial versions.

WPST trial version	Mean absolute error	SD	Min	Max		Percentiles			
					5th	25^th^	50^th^ median	75^th^	95th
Contralesional									
20-trial	15.2	7.7	4.3	42.4	5.3	9.2	13.6	20.4	27.8
10-trial	13.5	7.1	2.8	41.3	4.9	8.5	11.7	17.3	26.0
Ipsilesional									
20-trial	8.8	4.1	3.0	31.0	4.0	5.8	8.1	10.6	17.1
10-trial	8.1	4.0	1.8	27.8	3.3	5.2	7.5	9.8	16.3

*Note*: Scores are in absolute error, averaged for the number of test trials.

WPST: Wrist Position Sense Test; SD: standard deviation; Min: minimum; Max: maximum; score range for stroke survivors.

### Sensitivity and specificity of Wrist Position Sense Test: 10-trial version, relative to original 20-trial version

**Figure 3.**
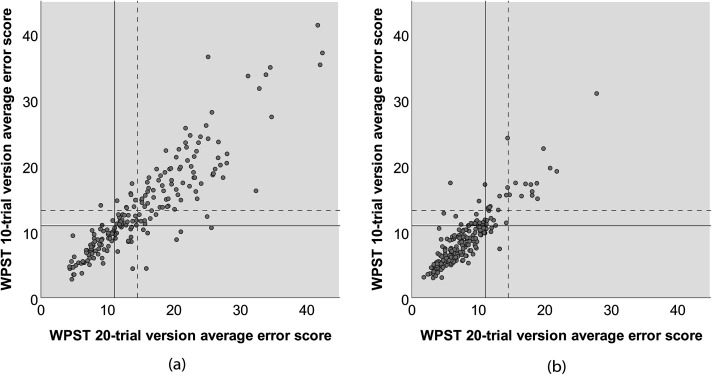
Scatterplot of the Wrist Position Sense Test (WPST) average error scores based on briefer 10-trial version when compared to 20-trial version for the (a) contralesional and (b) ipsilesional hands of the pooled stroke sample participants.

A scatterplot was used to display the spread and nature of the relationship between scores for the briefer 10-trial version and the 20-trial version (n = 205) for the contralesional hand of survivors of stroke. A positive association was found between the 20-trial and the 10-trial versions. The strength of the association was high (r = 0.89) (Figure 3(a)). The spread and nature of the relationship between both versions for the ipsilesional hand is also displayed (Figure 3(b)), with a similar high strength positive association of r = 0.88 for the 10-trial version.

**Figure 4.**
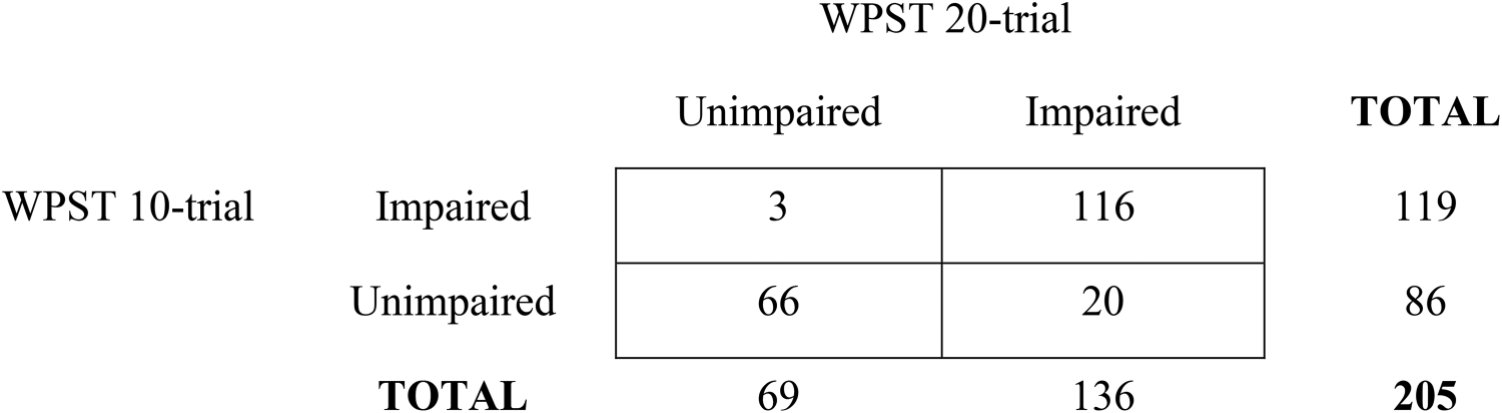
Contingency table analysis of contralesional limb position sense impairment identified using the Wrist Position Sense Test (WPST) 10-trial compared to the 20-trial version.

The presence and nature of agreement, or disagreement, in identification of contralesional wrist position sense impairment between test scores is indicated in the contingency table analysis (Figure 4). This analysis depicts the relationship between the 10-trial and 20-trial versions, and the occurrence of impairment when the corresponding 95th percentile criterion of abnormality is employed. The 10-trial compared to the 20-trial version had a sensitivity of 85.3% and specificity of 95.7%, based on scores for the contralesional hand.

## Discussion

We updated the normative standards and criteria of abnormality for the WPST in this pooled data study and evaluated a shorter, clinically feasible version, referred to as the WPST 10-trial version (WPST-10). Using pooled data from 298 participants (93 healthy adults and 205 stroke survivors), the new 95th percentile criterion for the original 20-trial version is 11.1^0^, and for the 10-trial version 10.9^0^. Age contributed minimally to the variance in WPST scores for both dominant and non-dominant hands of healthy participants and was not significant in our pooled healthy sample (n = 93). This finding supported combining the normative data across ages (i.e. 21 to 89 years) when interpreting scores. Similarly, hand dominance and sex also showed no significant differences, supporting a unified criterion in interpreting the WPST scores. Our findings support confident interpretation of proprioception impairment at the wrist relative to age-matched normative standards.

Using these updated criteria, 66% of survivors of stroke showed impairment in the contralesional (affected) hand and 21% in the ipsilesional (‘non-affected’) hand. The contralesional hand was more frequently and severely impaired than the ipsilesional hand, consistent with expectation and previous studies.^6,30,31^ Notably, 14.6% of participants with contralesional impairment also had impairment in the ipsilesional hand, supporting the notion that somatosensory disturbances post-stroke can affect both limbs.^6,32,33^ This highlights the importance of using quantitative clinical measures, such as the WPST, that do not confound response across limbs and assessing both limbs post-stroke using standardardised normative criteria to differentiate between normal and impaired wrist proprioception.

Updated normative and criterion of abnormality values based on a larger, pooled sample (n = 93 healthy; n = 205 stroke) can improve the clinical utility of the WPST, enabling accurate differentiation between normal and impaired wrist proprioceptive function. The best point estimate is 11.1^0^ mean error, with the pooled sample indicating that the true 95^th^ percentile could be between 10.3^0^ and 12.3^0^ for the 20-trial version, with a similar range for the WPST-10. These updated criterion of abnormality are consistent with earlier values.^3^ The updated norms now provide clinicians with clear cut-off scores for identifying wrist impairment, supporting more confident clinical decision-making and targeted rehabilitation interventions.

Quantification of proprioceptive impairment has been limited for this population to date. Quantitative assessments, like the KINARM, have shown that 67% of chronic stroke survivors experience proprioceptive loss.^7,10^ Our pooled analysis using the clinically oriented WPST aligns with this frequency (66%), although it should be recalled that our pooled sample included intervention trials in which survivors were clinically pre-screened for any somatosensory loss. These results continue to highlight the high number of survivors of stroke with proprioceptive impairment of the upper limb. Given the functional implications, assessing and treating this impairment should be considered a priority for rehabilitation.

The brief 10-trial version of the WPST may be considered an efficient and effective screen for identifying and quantifying wrist proprioceptive impairment, given its high sensitivity and specificity (85.3% and 95.7%, respectively), thus advancing current measurement of limb position sense for use within a clinical setting. Despite variability in assessors’ experience in the pooled sample, the 10-trial version maintained high accuracy, highlighting it as a robust test for routine clinical use. The WPST-10 has a low miss rate for identification of impairment in the contralesional upper limb (14.7%). This contrasts with the high miss rate of 66.7% for the contralesional upper limb when using routine clinical tests to screen relative to the original 20-trial version.^14^ Data from the Carey et al. (2002) study,^14^ which compared clinical and quantitative measures of proprioception, were also included in this current study, offering the opportunity to indirectly compare the detection rates of clinical testing with those using the WPST-10. The clinical test used in the earlier study^14^ was based on 10 clinician-positioned approximate wrist positions in flexion-extension range of movement. The inadequacy of current clinical measures in detecting impairments, further highlighted in the study by Kim and Choi-Kwon (1996) among acute stroke patients, underscores the importance of implementing quantitative and sensitive assessment methods in clinical practice.^32^

High detection rate and precision of the WPST-10 suggest its potential superiority over routine clinical assessments and supports its use in routine clinical practice as a screening tool. By enabling early and accurate identification of proprioceptive impairment, the WPST-10 can increase awareness of proprioceptive loss early, guide the development of targeted rehabilitation programs, facilitate monitoring of sensory recovery, and support evaluation of outcomes over time; a key premise of evidence-based stroke rehabilitation.

It is important to acknowledge the limitations of this study. First, findings from this study are limited to stroke survivors in sub-acute and chronic rehabilitation phases only. Participants were primarily from metropolitan Melbourne, English-speaking, and often pre-screened for somatosensory impairments, limiting generalisability. Future research should explore the implementation of WPST across a range of clinical practice settings and varying samples of stroke survivors to increase the generalisability of results. In addition, larger samples of healthy controls are needed to further investigate more subtle effects of age, sex, and hand dominance on WPST scores. It should be cautioned that an effect of age remains uncertain, given the relatively small sample size at the tail end of the distribution (95^th^ percentile) in the current pooled sample.

In conclusion, proprioceptive impairment is common after stroke, affecting both contralesional and ipsilesional wrists. The updated criteria of abnormality for the WPST now permit confident interpretation of scores in both research and clinical settings. Moreover, clinicians can be confident with identifying proprioceptive impairment of the wrist using the WPST-10. The WPST-10 may be used to screen for and detect impairment based on demonstrated high sensitivity and specificity relative to the reference original 20-trial version, supporting its use in clinical settings, contributing to improved identification, monitoring, and access to targeted intervention for proprioceptive impairment following stroke. With strong empirical and psychometric foundations, coupled with its efficiency in administration, it is recommended the WPST should supplement or replace existing clinical measures, which often fail to detect proprioceptive impairment.

## Clinical messages

- Updated criteria to identify impairment using the WPST will improve confident identification of wrist proprioceptive impairments in stroke survivors in clinical practice;
- The WPST-10 offers a screening alternative with high sensitivity and specificity, suitable for routine clinical use;
- Proprioceptive impairment in the contralesional wrist is common after stroke, with ipsilesional impairment also observed. It is recommended that the quantitative WPST, including WPST-10, be used in clinical practice settings to enhance identification of impairment and evidence-based practice.

## Supplemental Material

sj-docx-1-cre-10.1177_02692155251410469 - Supplemental material for Quantitative clinical assessment of wrist proprioception with stroke survivors Supplemental material, sj-docx-1-cre-10.1177_02692155251410469 for Quantitative clinical assessment of wrist proprioception with stroke survivors by Yvonne YK Mak-Yuen, Thomas A Matyas, Kylee Lockwood and Leeanne M Carey in Clinical Rehabilitation

## Data Availability

The data are not publicly available due to planned further analyses.
