# Supplementary material for: Quantitative clinical assessment of wrist proprioception with stroke survivors
Source: Clin Rehabil. 2026 Jan 5;40(4):498–508. doi: 10.1177/02692155251410469 (PMC13013658; doi:10.1177/02692155251410469)
Supplement: sj-docx-1-cre-10.1177_02692155251410469 - Supplemental material for Quantitative clinical assessment of wrist proprioception with stroke survivors [file sj-docx-1-cre-10.1177_02692155251410469.docx]

**Supplementary Table 1.** Demographic characteristics of stroke survivors and healthy controls according to pooled and individual study samples.

| Demographic  Information | Pooled  Sample | Discriminative  Validity ^a^ | SENSe  Trial ^b^ | IN_TOUCH Trial ^c^ | CoNNECT Trial ^d^ | NIH  linked  study ^e^ | SENSe  CONNECT ^f^ |
| --- | --- | --- | --- | --- | --- | --- | --- |
| **Stroke Survivors (n)** | 205 | 49 | 48 | 20 | 45 | 9 | 34 |
| Age, years |  |  |  |  |  |  |  |
| M (SD) | 57.7 (14.1) | 52 (14.6) | 59.7 (12.3) | 60.4 (15.3) | 52.8 (14.1) | 65.2 (12.6) | 56.2 (15.9) |
| Min, Max | 18, 87 | 18, 79 | 30, 87 | 18, 79 | 26, 82 | 41, 78 | 18,87 |
| Sex, n (%) |  |  |  |  |  |  |  |
| Male | 143 (69.8) | 35 (71) | 35 (73) | 13 (65) | 32 (71) | 6 (67) | 22 (65) |
| Female | 62 (30.2) | 14 (29) | 13 (27) | 7 (35) | 13 (29) | 3 (33) | 12 (35) |
| Lesion Level, n (%) |  |  |  |  |  |  |  |
| Cortical | 78 (38) | 15 (31) | 13 (27) | 8 (40) | 26 (58) | - | 16 (47) |
| Subcortical | 63 (31) | 9 (18) | 17 (35) | 12 (60) | 15 (33) | - | 10 (29) |
| Mixed | 24 (12) | 8 (16) | 11 (23) | 0 (0) | 4 (9) | - | 1 (3) |
| Unknown | 24 (12) | 17 (35) | 0 (0) | 0 (0) | 0 (0) | - | 7 (21) |
| Missing | 16 (8) | 0 (0) | 7 (15) | 0 (0) | 0 (0) | 9 (100) | 0 (0) |
| Stroke type, n (%) |  |  |  |  |  |  |  |
| Ischaemic | 120 (59) | 22 (45) | 30 (63) | 20 (100) | 31 (69) | - | 17 (50) |
| Haemorrhagic | 52 (25) | 7 (14) | 16 (33) | 0 (0) | 14 (31) | - | 15 (44) |
| Unknown | 22 (11) | 20 (41) | 0 (0) | 0 (0) | 0 (0) | - | 2 (6) |
| Missing | 11 (5) | 0 (0) | 2 (4) | 0 (0) | 0 (0) | 9 (100) | 0 (0) |
| Hemisphere affected,  n (%) |  |  |  |  |  |  |  |
| Right | 94 (46) | 20 (41) | 21 (44) | 7 (35) | 20 (45) | 5 (56) | 21 (62) |
| Left | 105 (51) | 28 (57) | 25 (52) | 13 (65) | 23 (51) | 4 (44) | 12 (35) |
| Both | 4 (2) | 1 (2) | 0 (0) | 0 (0) | 2 (4) | 0 (0) | 1 (3) |
| Missing | 2 (1) | 0 (0) | 2 (4) | 0 (0) | 0 (0) | 0 (0) | 0 (0) |
| Affected side, n (%) |  |  |  |  |  |  |  |
| Dominant | 103 (50) | 21 (43) | 30 (63) | 12 (60) | 23 (51) | 5 (56) | 12 (35) |
| Non dominant | 98 (48) | 24 (49) | 18 (37) | 8 (40) | 22 (49) | 4 (44) | 22 (65) |
| Missing | 4 (2) | 4 (8) | 0 (0) | 0 (0) | 0 (0) | 0 (0) | 0 (0) |
| **Healthy controls (n)** | 93 | 50 | N/A | N/A | 28 | 15 | N/A |
| Age, years |  |  |  |  |  |  |  |
| M (SD) | 51.5 (16.7) | 52.1 (12.9) | - | - | 50.0 (18.5) | 52.3 (23.9) | - |
| Min, Max | 21, 89 | 23, 77 | - | - | 26, 89 | 21, 82 | - |
| Sex, n (%) |  |  |  |  |  |  |  |
| Male | 58 (62.4) | 34 (68) | - | - | 16 (57.1) | 8 (53.3) | - |
| Female | 35 (37.6) | 16 (32) | - | - | 12 (42.9) | 7 (46.7) | - |
| Hand Dominance ^g^ n (%) |  |  |  |  |  |  |  |
| Right | 88 (94.6) | 46 (92) | - | - | 28 (100) | 14 (93.3) | - |
| Left | 5 (5.4) | 4 (8) | - | - | 0 (0) | 1 (6.7) | - |

Note: Total number of stroke participants = 205; Total number of healthy controls = 93.

SD = standard deviation

^a^ Stroke / Normative Discriminative Validity study [3]

^b^ SENSe = Study of Effectiveness of Neurorehabilitation on Sensation, intervention study [19]

^c^ IN_Touch = Imaging Neuroplasticity of Touch, neuroimaging with intervention study [20,21]

^d^ CoNNECT = Connecting Networks for Everyday Contact through Touch, neuroimaging with intervention study [22,23]

^e^ NIH linked study = stroke and healthy participants who underwent additional testing linked with the National Institute of Health Toolbox Study [24]

^f^ SENSe CONNECT Study, implementation study with intervention [25]

^g^ Based on Annette questionnaire of hand dominance or Edinburgh Handedness Inventory
